# Imaging of Cerebral Amyloid Angiopathy with Bivalent ^99m^Tc-Hydroxamamide Complexes

**DOI:** 10.1038/srep25990

**Published:** 2016-05-16

**Authors:** Shimpei Iikuni, Masahiro Ono, Hiroyuki Watanabe, Kenji Matsumura, Masashi Yoshimura, Hiroyuki Kimura, Hatsue Ishibashi-Ueda, Yoko Okamoto, Masafumi Ihara, Hideo Saji

**Affiliations:** 1 Department of Patho-Functional Bioanalysis, Graduate School of Pharmaceutical Sciences, Kyoto University, 46-29 Yoshida Shimoadachi-cho, Sakyo-ku, Kyoto 606-8501, Japan; 2 Department of Pathology, National Cerebral and Cardiovascular Center, 5-7-1 Fujishirodai, Suita-shi, Osaka 565-8565, Japan; 3 Department of Stroke and Cerebrovascular Diseases, National Cerebral and Cardiovascular Center, 5-7-1 Fujishirodai, Suita-shi, Osaka 565-8565, Japan

## Abstract

Cerebral amyloid angiopathy (CAA), characterized by the deposition of amyloid aggregates in the walls of cerebral vasculature, is a major factor in intracerebral hemorrhage and vascular cognitive impairment and is also associated closely with Alzheimer’s disease (AD). We previously reported ^99m^Tc-hydroxamamide (^99m^Tc-Ham) complexes with a bivalent amyloid ligand showing high binding affinity for β-amyloid peptide (Aβ(1–42)) aggregates present frequently in the form in AD. In this article, we applied them to CAA-specific imaging probes, and evaluated their utility for CAA-specific imaging. *In vitro* inhibition assay using Aβ(1–40) aggregates deposited mainly in CAA and a brain uptake study were performed for ^99m^Tc-Ham complexes, and all ^99m^Tc-Ham complexes with an amyloid ligand showed binding affinity for Aβ(1–40) aggregates and very low brain uptake. *In vitro* autoradiography of human CAA brain sections and *ex vivo* autoradiography of Tg2576 mice were carried out for bivalent ^99m^Tc-Ham complexes ([^99m^Tc]SB2A and [^99m^Tc]BT2B), and they displayed excellent labeling of Aβ depositions in human CAA brain sections and high affinity and selectivity to CAA in transgenic mice. These results may offer new possibilities for the development of clinically useful CAA-specific imaging probes based on the ^99m^Tc-Ham complex.

Cerebral amyloid angiopathy (CAA) is a sporadic or familial disorder characterized by the deposition of amyloid aggregates, mainly β-amyloid peptide (Aβ), in the walls of arteries and less often capillaries of the central nervous system, and belongs to the amyloidosis group^1^,^2^. CAA is present in 10–40% of the elderly^3^. In particular, at least a mild degree of CAA can be detected in up to 80% of patients with Alzheimer’s disease (AD)^3^, while severe CAA is present in approximately 25% of AD brains^4^.

CAA is a major cause of intracerebral hemorrhage (ICH) and vascular cognitive impairment^5^,^6^,^7^, and is also associated with small vessel diseases such as white matter hyperintensity and cerebral microbleeds^6^,^8^,^9^. CAA-associated ICH (CAA-ICH) comprises 5–20% of all spontaneous ICH in the elderly^1^,^3^. CAA-ICH is frequently a fatal condition and often recurs because of the multiple and widespread depositions of aggregated amyloid peptides in CAA brains^1^,^7^. Moreover, it was demonstrated that vascular diseases in the brain led to a decline of cognitive performance in the earliest stages of AD^10^,^11^.

Aβ(1–40) with a length of 40 amino acids is more soluble than longer Aβ(1–42) and is the main form in amyloid deposited in walls of blood vessels in CAA brains, while Aβ(1–42) is present more frequently in the form of senile plaque (SP) within the brain parenchyma in AD brains^1^,^2^. In patients with amyloidoses including CAA and AD, amyloid aggregates probably appear prior to onset of disease symptoms^12^,^13^; therefore, their detection *in vivo* may lead to an early diagnosis of the corresponding amyloidoses. Additionally, monitoring these amyloid aggregates *in vivo* may provide important information on the development of new medical techniques.

Brain biopsy is the gold standard for the diagnosis of CAA^7^; however, it is a highly invasive method. Although computed tomography (CT) and magnetic resonance imaging (MRI) are noninvasive and useful modalities for the diagnosis of CAA-ICH^14^,^15^, they detect intracerebral bleeding but not the deposition of amyloid aggregates; therefore, these indirect diagnostic techniques are unlikely to facilitate disease-specific diagnoses limited by the use of ICH as a surrogate marker for CAA. Accordingly, the development of a noninvasive technique to diagnose CAA-associated diseases specifically by the detection of amyloid using a probe is strongly needed.

Positron emission tomography (PET) and single photon emission computed tomography (SPECT) have generally been utilized as major *in vivo* imaging techniques to carry out the noninvasive diagnosis of amyloidoses. PET/SPECT can provide the information on localization of amyloid aggregates, while CT and MRI render the anatomical information. To date, many attempts to image Aβ aggregates constituting SP in AD brains using PET and SPECT tracers have been made. Several clinical studies using [^11^C]PIB, a neutral thioflavin-T analogue, have proved this utility for AD diagnosis^16^,^17^,^18^,^19^. More recently, [^18^F]florbetapir (Amyvid)^17^,^20^,^21^, [^18^F]flutemetamol (Vizamyl)^16^,^22^,^23^, and [^18^F]florbetaben (Neuraceq)^24^,^25^ have been approved by the US Food and Drug Administration for clinical AD diagnosis.

Similarly to SP in AD brains, there are several reports regarding the detection of cerebrovascular amyloid depositions using [^11^C]PIB^26^,^27^,^28^. However, since [^11^C]PIB is designed to penetrate the blood-brain barrier (BBB), it is considered to bind to not only vascular amyloid aggregates but also parenchymal amyloid aggregates, indicating that it detects amyloid depositions in the whole brain; therefore, [^11^C]PIB cannot help detecting SP as background signal in case of the diagnosis of CAA. Several efforts toward the development of imaging probes targeting Aβ deposition in CAA have been made. These probes, designed as fluorescent dye^29^, MRI contrast^30^,^31^,^32^, or PET/SPECT imaging^31^,^32^,^33^,^34^ agents, showed a potential use for CAA; however, *in vivo* specificity for Aβ aggregates in CAA was not demonstrated. Further research into the development of imaging probes for selective binding to Aβ deposited in the walls of the cerebral vasculature and to differentiate CAA from AD is desired.

To detect CAA but not SP, low brain uptake of an imaging probe targeting Aβ aggregates may be favorable^33^,^34^. We previously reported a series of ^99m^Tc-hydroxamamide (^99m^Tc-Ham) complexes with a multivalent amyloid ligand^35^, and utilized stilbene (SB) and benzothiazole (BT) as ligands for amyloid aggregates. These compounds are believed to hardly cross the BBB *in vivo*, and their high binding affinity for Aβ aggregates is feasible for imaging CAA. However, in that report, the binding affinity of ^99m^Tc-Ham complexes was evaluated using Aβ(1–42) aggregates present mainly in SP and less often CAA. It is generally accepted that compounds with high binding affinity for Aβ(1–42) aggregates except for antibodies can bind to other amyloid aggregates such as tau and α-synuclein^36^,^37^,^38^. Therefore, ^99m^Tc-Ham complexes are considered to bind to Aβ(1–40) aggregates, the predominant amyloid found in CAA, similarly to Aβ(1–42) aggregates.

In the present study, we evaluated the binding affinity for Aβ(1–40) aggregates deposited mainly in CAA of ^99m^Tc-Ham complexes with a monovalent or bivalent amyloid ligand ([^99m^Tc]SB1, [^99m^Tc]SB2, [^99m^Tc]BT1, and [^99m^Tc]BT2) (Fig. 1), and their utility for the *in vivo* specific detection of vascular amyloid aggregates but not parenchymal amyloid aggregates.

## Results

### Synthesis and ^99m^Tc labeling

The ^99m^Tc labeling reaction was performed by the complexation reaction using the Ham precursor, ^99m^Tc-pertechnetate, and tin (II) tartrate hydrate as a reducing agent^35^. The ^99m^Tc complexation reaction with Ham derivatives provided two isomers of ^99m^Tc-Ham complexes, as described in previous reports^35^,^39^. We defined the specific isomers with shorter retention times on reversed-phase high-performance liquid chromatography (RP-HPLC) as A-form ([^99m^Tc]SB1A, [^99m^Tc]SB2A, [^99m^Tc]BT1A, and [^99m^Tc]BT2A), and the others as B-form ([^99m^Tc]SB1B, [^99m^Tc]SB2B, [^99m^Tc]BT1B, and [^99m^Tc]BT2B).

### ^99m^Tc-Ham complexes showed high binding affinity for Aβ(1–40) aggregates in solution

To evaluate binding affinity for Aβ(1–40) aggregates of ^99m^Tc-Ham complexes, we performed an inhibition binding assay with PIB as a competitive ligand. A fixed concentration of Aβ(1–40) aggregates and the ^99m^Tc-Ham complex were incubated with increasing concentrations of nonradioactive PIB. PIB showed IC_50_ values of 0.38, 0.45, 4.59, 3.37, 0.24, 0.99, 1.58, and 4.96 μM in the presence of [^99m^Tc]SB1A, [^99m^Tc]SB1B, [^99m^Tc]SB2A, [^99m^Tc]SB2B, [^99m^Tc]BT1A, [^99m^Tc]BT1B, [^99m^Tc]BT2A, and [^99m^Tc]BT2B, respectively (Table 1).

### Assessment of BBB permeability

**Figure 2:**
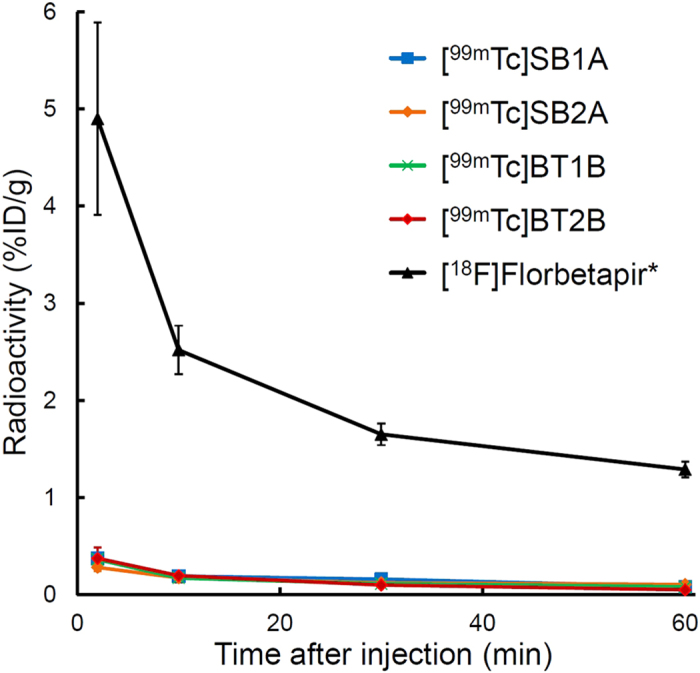
Comparison of radioactivity of extracted brain tissues after intravenous injection of ^99m^Tc-Ham complexes and [^18^F]florbetapir in normal mice. Values are the mean ± standard deviation of 5 animals. *Data from our previous article (ref. 45).

To evaluate brain uptake of ^99m^Tc-Ham complexes, biodistribution experiments of ^99m^Tc-Ham complexes were performed in normal mice. We selected ^18^F-florbetapir as a control and compared the results of ^99m^Tc-Ham complexes with that of ^18^F-florbetapir (Fig. 2). The brain uptake of [^99m^Tc]SB1A, [^99m^Tc]SB2A, [^99m^Tc]BT1B, and [^99m^Tc]BT2B at 2 min postinjection was 0.37, 0.28, 0.36, and 0.37% injected dose (ID)/g, respectively. The radioactivity in the brains remained low until 60 min postinjection. The results of the biodistribution study are shown in Table S1 in Supplementary information.

### ^99m^Tc-Ham complexes including bivalent amyloid ligand displayed excellent labeling of Aβ depositions in human CAA brain sections

**Figure 3:**
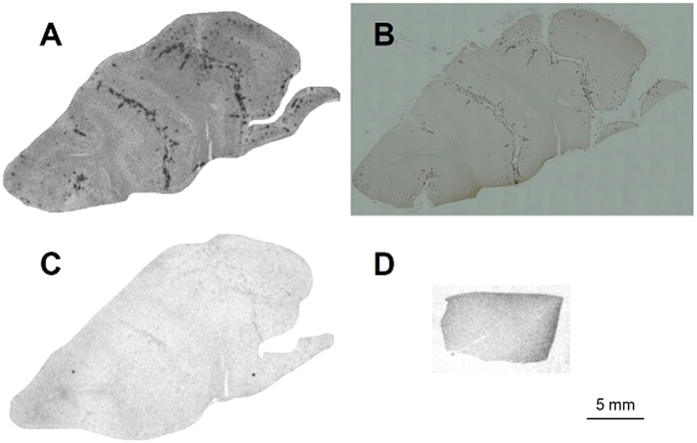
*In vitro* autoradiogram of a brain section from a patient with CAA (female, 67 years old) labeled with [^99m^Tc]SB2A (**A**). The same brain section was immunostained with an antibody against Aβ(1–40) (**B**). Blocking study with nonradioactive PIB was also performed using the adjacent brain section (**C**). *In vitro* autoradiogram of a brain section from a healthy control (male, 73 years old) labeled with [^99m^Tc]SB2A (**D**).

The binding of [^99m^Tc]SB2A and [^99m^Tc]BT2B to Aβ depositions in brain sections from a CAA patient was evaluated by *in vitro* autoradiography. In CAA brain sections, [^99m^Tc]SB2A intensively labeled Aβ depositions (Fig. 3A), while almost no accumulation of radioactivity was observed in the control brain sections (Fig. 3D). Furthermore, the labeling pattern was consistent with the immunohistochemical staining pattern observed in the same brain sections with anti-Aβ(1–40) antibody (Fig. 3B). In addition, the labeling of Aβ depositions with [^99m^Tc]SB2A was blocked to a large extent with an excess of nonradioactive PIB (Fig. 3C). *In vitro* autoradiography with [^99m^Tc]BT2B showed a similar result to that of [^99m^Tc]SB2A (Fig. S1 in Supplementary information). Moreover, [^99m^Tc]SB2A and [^99m^Tc]BT2B also intensively labeled Aβ depositions in brain sections from another patient with CAA (Fig. S2 in Supplementary information).

### ^99m^Tc-Ham complexes including bivalent amyloid ligand exhibited high affinity and selectivity to CAA in transgenic mice

**Figure 4:**
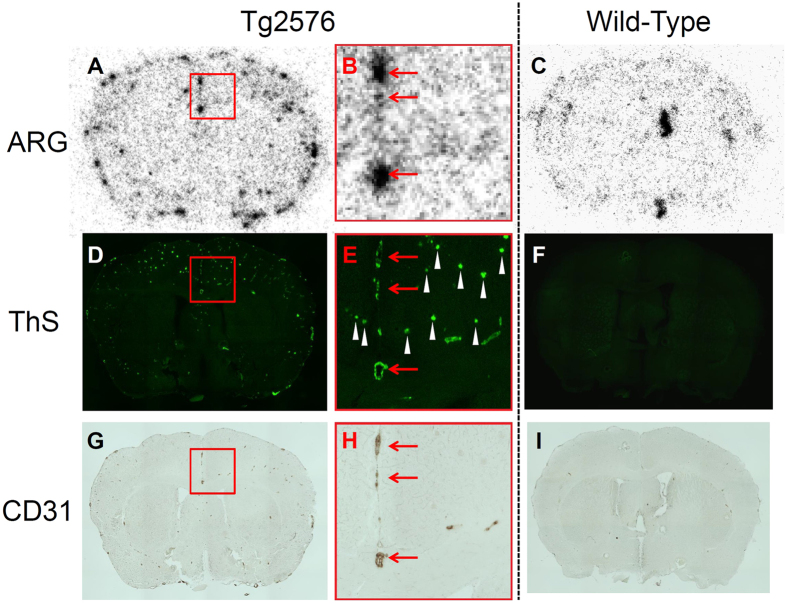
*Ex vivo* autoradiograms (ARG) from Tg2576 (**A**) and wild-type (**C**) mice with [^99m^Tc]SB2A. The same sections were stained with thioflavin-S (ThS) (**D,F**). The same sections were also immunostained with an antibody against CD31 (**G,I**). Panel B,E,H represent magnified image details of panel A,D,G, respectively. Red arrows show Aβ depositions labeled with both ThS and anti-CD31 antibody. White arrowheads show Aβ depositions labeled with ThS, not anti-CD31 antibody.

To confirm the affinity of [^99m^Tc]SB2A and [^99m^Tc]BT2B for Aβ aggregates in a mouse brain, *ex vivo* autoradiography was performed using Tg2576 and wild-type mice (Fig. 4). The brains were removed at 30 min postinjection for autoradiography. *Ex vivo* autoradiograms with [^99m^Tc]SB2A displayed intensive labeling of Aβ depositions in the transgenic mice (Fig. 4A,B) but not the age-matched controls (Fig. 4C). The labeling pattern on autoradiograms was partially consistent with the staining pattern observed in the same brain sections from Tg2576 mice with thioflavin-S, a dye commonly used to stain Aβ depositions (Fig. 4D,E), while there was no marked staining in the wild-type mouse brain sections (Fig. 4F). However, some Aβ depositions were not labeled with [^99m^Tc]SB2A in Tg2576 mouse brain sections. To confirm whether [^99m^Tc]SB2A labeled Aβ aggregates deposited within vascular or parenchyma, the same sections were immunostained with anti-CD31 antibody, a marker for endothelial cells (Fig. 4G–I)^40^,^41^. The accumulation of radioactivity on autoradiograms was observed only at Aβ depositions labeled with both thioflavin-S and anti-CD31 antibody (Fig. 4B,E and H, red arrows), while no radioactive spots were observed at Aβ depositions labeled with thioflavin-S, not anti-CD31 antibody (Fig. 4E, white arrowheads). Furthermore, *ex vivo* autoradiography with [^99m^Tc]BT2B showed a similar result to that of [^99m^Tc]SB2A (Fig. S3 in Supplementary information).

## Discussion

We previously reported ^99m^Tc-Ham complexes with a bivalent amyloid ligand showing high binding affinity for Aβ(1–42) aggregates^35^. In the present study, we evaluated their utility as CAA-specific imaging probes. Recently, several new ^99m^Tc-labeled CAA imaging agents were reported^42^,^43^,^44^. In spite of the fact that they were synthesized under heating and acidic conditions, ^99m^Tc-Ham complexes can be prepared under mild conditions (non-heating and neutral), indicating that ^99m^Tc-Ham complexes may be superior to the other CAA-imaging probes reported previously. *In vitro* binding study using Aβ(1–40) aggregates, a major form of amyloid in CAA, exhibited that the amyloid ligand dimers ([^99m^Tc]SB2 and [^99m^Tc]BT2) bound to Aβ(1–40) aggregates more strongly than their monomers ([^99m^Tc]SB1 and [^99m^Tc]BT1), and A-form of ^99m^Tc-Ham complexes with BT derivatives showed lower binding affinity than B-form, as demonstrated in our previous report using Aβ(1–42) aggregates (Table 1). However, specific isomers of ^99m^Tc-Ham complexes with SB derivatives displayed similar binding affinity for Aβ(1–40) aggregates to those of the other isomers, while significant differences between IC_50_ values with two isomers of SB derivatives were shown in the inhibition assay using Aβ(1–42) aggregates^35^. All ^99m^Tc-Ham complexes showed blocked binding to amyloid aggregates with a very high concentration (μM order) of PIB, although the Aβ imaging probes reported previously have a binding affinity equal to or lower than that of PIB, suggesting that they have a much higher binding affinity than any other tracers targeting Aβ including CAA-specific imaging probes^44^. Among eight ^99m^Tc-Ham complexes, both [^99m^Tc]SB2A and [^99m^Tc]BT2B showed high binding affinity for Aβ(1–40) aggregates (4.59 and 4.96 μM, respectively), which was higher than those of any other ^99m^Tc-Ham complexes.

According to the result of the inhibition assay using Aβ(1–40) and Aβ(1–42) aggregates, brain uptake studies were performed for only specific isomers with the higher binding affinity for Aβ aggregates (A-form of SB derivatives and B-form of BT derivatives). [^99m^Tc]SB1A, [^99m^Tc]SB2A, [^99m^Tc]BT1B, and [^99m^Tc]BT2B displayed much lower initial brain uptake than [^18^F]florbetapir under similar experimental conditions (4.90%ID/g at 2 min postinjection) (Fig. 2)^45^, while [^18^F]florbetapir has proved its utility for imaging Aβ plaques in the brain. In addition, ^99m^Tc-Ham complexes also showed a lower initial brain entry than even other CAA imaging probes reported previously (0.61–1.21%ID/g at that time)^44^. These results suggest that ^99m^Tc-Ham complexes could hardly cross the BBB. Therefore, they may be incapable of binding to Aβ aggregates deposited within the brain parenchyma. According to the results of binding affinity for Aβ(1–40) and Aβ(1–42) aggregates *in vitro* and brain uptake in normal mice *ex vivo*, further studies were conducted using [^99m^Tc]SB2A and [^99m^Tc]BT2B with high binding affinity for Aβ aggregates and very low brain uptake.

*In vitro* autoradiography of human CAA brain sections with [^99m^Tc]SB2A showed intensive labeling of Aβ depositions (Fig. 3A), confirmed by immunostaining of the same brain sections with anti-Aβ(1–40) antibody (Fig. 3B). Many ^99m^Tc-labeled Aβ imaging probes with preferable binding affinity have exhibited no marked labeling of Aβ depositions in human brain sections; however, Jia *et al*. recently reported a ^99m^Tc-labeled tracer showing positive autoradiography results for brain sections from AD patients^44^. As well as those results, ^99m^Tc-Ham complexes showed excellent labeling of Aβ depositions in human brain sections. Additionally, a blocking study with nonradioactive PIB confirmed the specific binding of [^99m^Tc]SB2A to Aβ depositions in CAA brain sections (Fig. 3C). *In vitro* autoradiography with [^99m^Tc]BT2B showed specific binding to Aβ depositions in CAA brain sections as well as [^99m^Tc]SB2A (Fig. S1 in Supplementary information). In addition, two bivalent ^99m^Tc-Ham complexes, [^99m^Tc]SB2A and [^99m^Tc]BT2B, also showed intensive labeling of Aβ depositions in brain sections from another patient with CAA (Fig. S2 in Supplementary information).

*Ex vivo* autoradiography with [^99m^Tc]SB2A displayed specific binding to Aβ aggregates in the living Tg2576 mouse brain (Fig. 4A,B) but not wild-type mouse brain (Fig. 4C). Since Tg2576 mice are known to overproduce Aβ aggregates in the brain, they have been commonly used to evaluate the specific binding of Aβ aggregates in experiments *in vitro* and *in vivo*^45^,^46^. The accumulation of radioactivity in Tg2576 mouse brain sections was observed only at the presence of both amyloid aggregates (Fig. 4D,E) and endothelial cells (Fig. 4G,H), suggesting that [^99m^Tc]SB2A selectively bound to amyloid aggregates deposited along vessels but not within parenchyma. In addition, [^99m^Tc]BT2B displayed specific detection of CAA in Tg2576 mice (Fig. S3 in Supplementary information). These results are consistent with the biodistribution study showing the low brain entry of ^99m^Tc-Ham complexes. These findings in the present study suggest that our bivalent ^99m^Tc-Ham complexes, [^99m^Tc]SB2A and [^99m^Tc]BT2B, can specifically detect CAA *in vivo*. However, these tracers seemed to label areas in the cortex that are not apparent in the thioflavin-S staining. Thioflavin-S has much lower affinity (K_d_: μM order) than useful Aβ imaging probes reported previously such as PIB, florbetapir (K_d_: nM order)^19^,^47^. An *in vitro* inhibition assay showed that our ^99m^Tc-labeled compounds blocked binding to Aβ aggregates due to a much higher concentration of unlabeled-PIB, suggesting that our compounds have a much higher binding affinity than other Aβ and CAA imaging agents reported previously. Accordingly, it is considered that thioflavin-S can detect fewer depositions of amyloid than our compounds. In addition, we also carried out *ex vivo* autoradiography using perfused mouse brains, and obtained results showing differences with Tg2576 and wild-type mice (data not shown), suggesting that radioactivity was derived from tracers binding to depositions of amyloid, and not from tracers contained in the blood. Although the possibility that the BBB is leaky in the brains of AD patients has been suggested in several reports^48^,^49^, it has remained controversial whether or not the BBB dysfunction depends on the stage of AD. Not all studies have indentified an index of BBB disruption in AD brains^50^,^51^,^52^, but Zipser *et al*. recently reported that dysfunction of the BBB could increase stepwisely with the degree of pathology in AD^53^, indicating that the BBB should be intact in an early stage of preclinical AD. Moreover, CAA imaging probes should be used in the preclinical stage of the disease when the BBB functions normally. Therefore, ^99m^Tc-Ham complexes developed in the present study, which may be incapable of penetrating the BBB, can serve as CAA-specific imaging probes for the early diagnosis of AD.

In addition, we performed an *in vivo* SPECT/CT study with [^99m^Tc]SB2A using Tg2576 and wild-type mice at 30 min postinjection (Fig. S4 in Supplementary information). Although *ex vivo* autoradiography demonstrated the specificity of [^99m^Tc]SB2A for CAA, [^99m^Tc]SB2A was not differentially distributed in the brains of Tg2576 and wild-type mice *in vivo*. The radioactivity accumulation was observed mostly in the limbic region of the brain, which seemed to be derived from the blood. This inference is supported by the observations that blood vessels were rich in this region of the brain confirmed by immunostaining of CD31 (Fig. 4G,I), and a biodistribution study which suggested that [^99m^Tc]SB2A has a high retention rate in the blood (5.13%ID/g at 30 min postinjection, Table S1 in Supplementary information). Although the influence of radioactivity in the cerebral blood could be removed by perfusion in *ex vivo* autoradiography examination, it was inevitable to detect radioactivity in the blood as a background signal in the *in vivo* SPECT study. Furthermore, we carried out an *in vivo* SPECT imaging study at a later time point (120 min postinjection) of [^99m^Tc]SB2A. However, we obtained a similar result to that of at 30 min postinjection, suggesting that the radioactivity in the blood still remained at 120 min postinjection (Fig. S5 in Supplementary information). Therefore, further acceleration of the clearance of ^99m^Tc-labeled probes from the blood pool is essential for the development of *in vivo* imaging probes targeting CAA. The introduction of a hydrophilic substituted group including hydroxyl and carboxyl groups may constitute one of the strategies to enhance the clearance of probes from the blood. For instance, the replacement of the dimethylamino group in [^99m^Tc]SB2A with a hydroxyl group reduces its lipophilicity, contributing to lower binding to plasma proteins. This modification of probes should facilitate the more rapid clearance of [^99m^Tc]SB2A from the blood, leading to a lower background signal that can bring about an increase in the specific signal of the probes on binding to CAA.

In the current study, we applied bivalent ^99m^Tc-Ham complexes that we reported previously to imaging probes targeting CAA. All ^99m^Tc-Ham complexes including a monovalent or bivalent amyloid ligand showed binding affinity for Aβ(1–40) aggregates *in vitro* and very low brain uptake in normal mice *ex vivo. In vitro* autoradiography showed specific binding of ^99m^Tc-Ham complexes including a bivalent amyloid ligand ([^99m^Tc]SB2A and [^99m^Tc]BT2B) with high binding affinity in the inhibition assay to Aβ depositions in brain sections from a CAA patient. Additionally, [^99m^Tc]SB2A and [^99m^Tc]BT2B displayed excellent and selective labeling of Aβ depositions in vessels but not parenchyma in mouse brains. The results suggest that [^99m^Tc]SB2A and [^99m^Tc]BT2B have potential as CAA-specific imaging probes. Although the *in vivo* SPECT/CT study with [^99m^Tc]SB2A showed no marked difference in radioactivity accumulation in the brain between Tg2576 and wild-type mice, the findings in the present study reveal new possibilities of developing clinically useful CAA imaging probes based on the ^99m^Tc-Ham complex. Further optimization to improve the clearance of ^99m^Tc-Ham complexes from the blood is underway.

## Methods

### General

All reagents were obtained commercially and used without further purification unless otherwise indicated. PIB was purchased from ABX (Saxony, Germany). Na^99m^TcO_4_ was purchased from Nihon Medi-Physics Co., Ltd. (Tokyo, Japan) or was obtained from a commercial ^99^Mo/^99m^Tc generator (Ultra-Techne Kow; FUJIFILM RI Pharma Co., Ltd., Tokyo, Japan). RP-HPLC was performed with a Shimadzu system (SHIMADZU, Kyoto, Japan, a LC-20AT pump with a SPD-20A UV detector, λ = 254 nm) with a Cosmosil C_18_ column (Nacalai Tesque, Kyoto, Japan, 5C_18_-AR-II, 4.6 mm × 150 mm) using a mobile phase (10 mM phosphate buffer (pH 7.4)/acetonitrile: 0 min 3/2 to 30 min 3/7) delivered at a flow rate of 1.0 mL/min.

### Animals

Animal experiments were conducted in accordance with our institutional guidelines and were approved by the Kyoto University Animal Care Committee. Male ddY mice were purchased from Japan SLC, Inc. (Shizuoka, Japan). Female Tg2576 mice and wild-type mice were purchased from Taconic Farms, Inc. (New York, USA). Animals were fed standard chow and had free access to water. All efforts were made to minimize suffering.

### Human brain tissues

Experiments involving human subjects were performed in accordance with relevant guidelines and regulations and were approved by the ethics committee of Kyoto University and National Cerebral and Cardiovascular Center. Informed consent was secured from all subjects in this study. Postmortem brain tissues from autopsy-confirmed cases of CAA (female, 67 years old, and female, 85 years old) and a control (male, 73 years old) were obtained from the Graduate School of Medicine, Kyoto University, National Cerebral and Cardiovascular Center, and BioChain Institute, Inc. (California, USA), respectively.

### Synthesis and ^99m^Tc labeling

^99m^Tc-Ham complexes ([^99m^Tc]SB1, [^99m^Tc]SB2, [^99m^Tc]BT1, and [^99m^Tc]BT2) were prepared as we reported previously^35^. In brief, to solutions of 0.2 mg Ham precursors ((*Z*)-4-((*E*)-4-(dimethylamino)styryl)-*N’*-hydroxybenzimidamide, (*Z*)-2-(4-(dimethylamino)phenyl)-*N’*-hydroxybenzo[*d*]thiazole-6-carboximidamide, and (*Z*)-4-(dimethylamino)-*N’*-hydroxybenzimidamide) in acetate/ethanol (1/4, 200 μL) were added 100 μL Na^99m^TcO_4_ solution and 15 μL tin (II) tartrate hydrate solution [2 mg tin (II) tartrate hydrate (7.50 μmol) dissolved in water (2.5 mL)]. The reaction mixtures were incubated at room temperature for 30 min and purified by RP-HPLC. The ^99m^Tc-Ham complexes were analyzed by analytical RP-HPLC on a Cosmosil C_18_ column (5C_18_-AR-II, 4.6 mm × 150 mm) with a solvent of phosphate buffer (10 mM, pH 7.4)/acetonitrile (0 min 3/2 to 30 min 3/7) as the mobile phase at a flow rate of 1.0 mL/min. The radioactivity of the ^99m^Tc-labeled compounds was recorded for 30 min.

### Competitive inhibition assay using Aβ(1–40) aggregates in solution

A solid form of Aβ(1–40) was purchased from the Peptide Institute (Osaka, Japan). Aggregation was carried out by gently dissolving the peptide (0.50 mg/mL) in phosphate-buffered saline (PBS) (pH 7.4). The solution was incubated at 37 °C for 42 h with gentle and constant shaking. A mixture containing 50 μL Aβ(1–40) aggregates (final conc., 1.25 μg/mL), 50 μL ^99m^Tc-Ham complex (final conc., 8.3 kBq/mL), 50 μL PIB (final conc., 64 pM–125 μM in 30% EtOH), and 850 μL of 30% EtOH was incubated at room temperature for 3 h. The mixture was filtered through Whatman GF/B filters (Whatman, Kent, U.K.) using a Brandel M-24 cell harvester (Brandel, Maryland, USA), and the radioactivity of the filters containing the bound ^99m^Tc-Ham complex was measured using a γ counter (Wallac 1470 Wizard; PerkinElmer, Massachusetts, USA). Values for the half-maximal inhibitory concentration (IC_50_) were determined from displacement curves using GraphPad Prism 5.0 (GraphPad Software, Inc., California, USA).

### *Ex vivo* biodistribution in normal mice

A saline solution (100 μL) of ^99m^Tc-Ham complexes (20 kBq) containing EtOH (10 μL) was injected directly into the tail vein of ddY mice (male, 5 weeks old). The mice were sacrificed at 2, 10, 30, and 60 min postinjection. The organs of interest were removed and weighed, and radioactivity was measured using a γ counter (PerkinElmer). The %ID/g of samples was calculated by comparing the sample counts with the count of the diluted initial dose.

### *In vitro* autoradiography of human CAA brain sections

Six micrometer thick serial human brain sections of paraffin-embedded blocks were used for autoradiography. To completely deparaffinize the sections, they were incubated in xylene for 30 min two times and in 100% EtOH for 1 min two times. Subsequently, they were subjected to 1-min incubation in 90% EtOH and 1-min incubation in 70% EtOH, followed by a 5-min wash in water. Each slide was incubated with a 50% EtOH solution of [^99m^Tc]SB2A or [^99m^Tc]BT2B (370 kBq/mL) at room temperature for 1 h. For blocking experiments, the adjacent sections were incubated with a 50% EtOH solution of [^99m^Tc]SB2A or [^99m^Tc]BT2B (370 kBq/mL) in the presence of nonradioactive PIB (1.0 mM). The sections were washed in 50% EtOH for 3 min two times and exposed to a BAS imaging plate (Fuji Film, Tokyo, Japan) for 2 h. Autoradiographic images were obtained using a BAS5000 scanner system (Fuji Film). After autoradiographic examination, the same sections were immunostained by an antibody against Aβ(1–40) to confirm the presence of Aβ depositions. For immunohistochemical staining of Aβ(1–40), the sections were autoclaved for 15 min in 0.01 M citric acid buffer (pH 6.0) to activate the antigen. After three 5-min incubations in PBS-Tween 20 (PBST), they were incubated with anti-Aβ(1–40) primary antibody (BA27; Wako, Osaka, Japan) at room temperature overnight. Subsequently, they were incubated in PBST for 5 min three times, and incubated with biotinylated goat anti-mouse IgG (Wako) at room temperature for 3 h. After three 5-min incubations in PBST, the sections were incubated with Streptavidin-Peroxidase complex at room temperature for 30 min. After three 5-min incubations in PBST, they were incubated with diaminobenzidine (Merck, Hesse, Germany) as a chromogen for 5 min. After washing with water, the sections were observed under a microscope (BIOREVO BZ-9000; Keyence Corp., Osaka, Japan).

### *Ex vivo* autoradiography using Tg2576 and wild-type mice

Tg2576 transgenic mice (female, 29 months old) and wild-type mice (female, 29 months old) were used as the AD model and age-matched control, respectively. A saline solution (150 μL) of [^99m^Tc]SB2A or [^99m^Tc]BT2B (18.5 MBq) containing EtOH (30 μL) was injected through the tail vein. The mice were sacrificed at 30 min postinjection. The brains were immediately removed, embedded in carboxymethylcellulose solution and then frozen in a dry ice/hexane bath. Sections of 30 μm were cut and exposed to a BAS imaging plate (Fuji Film) overnight. Autoradiographic images were obtained using a BAS5000 scanner system (Fuji Film). After autoradiographic examination, the same sections were stained by thioflavin-S to confirm the presence of Aβ depositions. For thioflavin-S fluorescent staining, the sections were immersed in a 100 μM thioflavin-S solution containing 50% EtOH for 3 min, washed in 50% EtOH for 1 min two times, and examined using a microscope (Keyence Corp.) equipped with a GFP-BP filter set. Additionally, the same sections were immunostained by anti-CD31 antibody to confirm the presence of endothelial cells. For immunohistochemical staining of CD31, the sections were incubated in PBST for 5 min three times, and incubated with anti-CD31 primary antibody (SZ31; Abcam, Cambridgeshire, U.K., dilution 1:50) at room temperature overnight. After three 5-min incubations in PBST, anti-rabbit secondary antibody (Dako, California, USA) incubation was carried out at room temperature for 3 h. Subsequently, the sections were incubated in PBST for 5 min three times, and incubated with diaminobenzidine (Merck) as a chromogen for 5 min. After washing with water, the sections were observed under a microscope (Keyence Corp.).

## Additional Information

**How to cite this article**: Iikuni, S. *et al*. Imaging of Cerebral Amyloid Angiopathy with Bivalent ^99m^Tc-Hydroxamamide Complexes. *Sci. Rep.* **6**, 25990; doi: 10.1038/srep25990 (2016).

## Supplementary Material

Supplementary Information

## Figures and Tables

**Figure 1:**
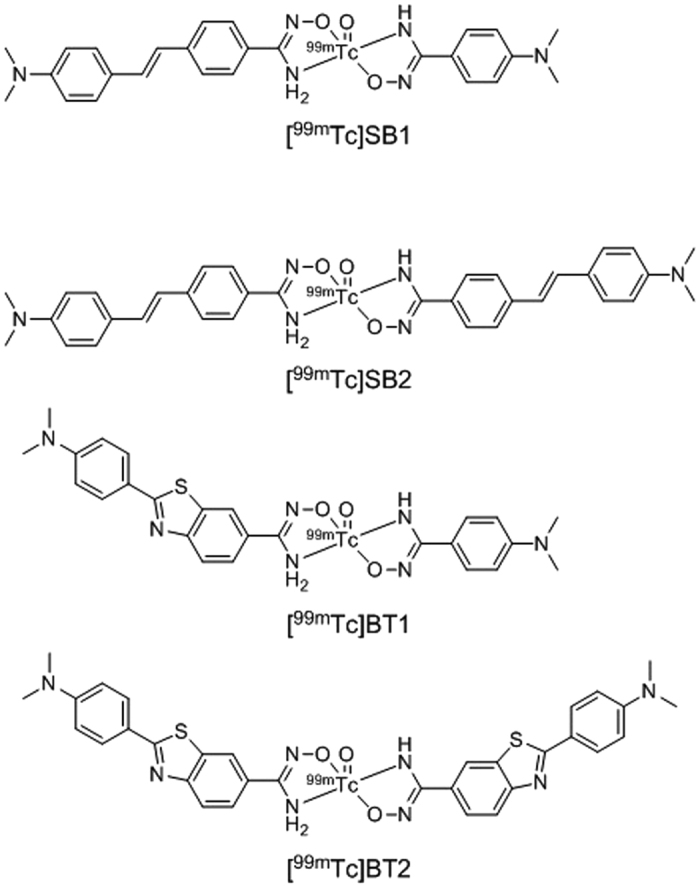
Proposed structure of the ^99m^Tc-Ham complexes.

**Table 1:** Half-maximal inhibitory concentration (IC_50_, μM) for the binding of PIB to Aβ aggregates determined using ^99m^Tc-Ham complexes as ligands.

Compound	IC_50_ of PIB (μM)*	
	Aβ(1–40)	Aβ(1–42)†
[^99m^Tc]SB1A	0.38 ± 0.06	0.72 ± 0.10
[^99m^Tc]SB1B	0.45 ± 0.11	0.38 ± 0.08
[^99m^Tc]SB2A	4.59 ± 0.77	16.40 ± 2.47
[^99m^Tc]SB2B	3.37 ± 0.61	2.55 ± 0.45
[^99m^Tc]BT1A	0.24 ± 0.06	0.26 ± 0.02
[^99m^Tc]BT1B	0.99 ± 0.17	0.47 ± 0.05
[^99m^Tc]BT2A	1.58 ± 0.27	2.80 ± 0.32
[^99m^Tc]BT2B	4.96 ± 0.90	5.78 ± 0.53

^*^Values are the mean ± standard error of the mean of 6–15 independent experiments.

^†^Data from our previous article (ref. 35).
