# Supplementary material for: Imaging of Cerebral Amyloid Angiopathy with Bivalent 99mTc-Hydroxamamide Complexes
Source: Sci Rep. 2016 May 16;6:25990. doi: 10.1038/srep25990 (PMC4867616; doi:10.1038/srep25990)
Supplement: Supplementary Information [file srep25990-s1.doc]

**SUPPLEMENTARY INFORMATION**

**Imaging of Cerebral Amyloid Angiopathy with Bivalent ^99m^Tc-Hydroxamamide Complexes**

Shimpei Iikuni^1^, Masahiro Ono*^,1^, Hiroyuki Watanabe^1^, Kenji Matsumura^1^, Masashi Yoshimura^1^, Hiroyuki Kimura^1^, Hatsue Ishibashi-Ueda^2^, Yoko Okamoto^2^, Masafumi Ihara^3^ & Hideo Saji^1^

^1^Department of Patho-Functional Bioanalysis, Graduate School of Pharmaceutical Sciences, Kyoto University, 46-29 Yoshida Shimoadachi-cho, Sakyo-ku, Kyoto 606-8501, Japan, ^2^Department of Pathology, National Cerebral and Cardiovascular Center, 5-7-1 Fujishirodai, Suita-shi, Osaka 565-8565, Japan, ^3^Department of Stroke and Cerebrovascular Diseases, National Cerebral and Cardiovascular Center, 5-7-1 Fujishirodai, Suita-shi, Osaka 565-8565, Japan.

*Corresponding author: M. Ono. Address: 46-29 Yoshida Shimoadachi-cho, Sakyo-ku, Kyoto 606-8501, Japan, Phone: +81-75-753-4608, Fax: +81-75-753-4568, E-mail: ono@pharm.kyoto-u.ac.jp.

***Ex vivo* biodistribution in normal mice.** A saline solution (100 μL) of ^99m^Tc-Ham complexes (20 kBq) containing EtOH (10 μL) was injected directly into the tail vein of ddY mice (male, 5 weeks old). The mice were sacrificed at 2, 10, 30, and 60 min postinjection. The organs of interest were removed and weighed, and radioactivity was measured using a γ counter (Wallac 1470 Wizard; PerkinElmer, Massachusetts, USA). Percentage dose per organ was calculated by comparing the tissue counts to suitably diluted aliquots of the injected material. The %dose/g of samples was calculated by comparing the sample counts with the count of the diluted initial dose.

**Table S1. Biodistribution of radioactivity after intravenous injection of ^99m^Tc-Ham complexes in normal mice***

| Tissue | Time after injection (min) | | | | | | |
| --- | --- | --- | --- | --- | --- | --- | --- |
|  | 2 | | 10 | | 30 | | 60 |
| [^99m^Tc]SB1A | | | | | | | |
| Blood | 18.89 (4.57) | 9.30 (1.96) | | 6.06 (0.29) | | 2.71 (0.14) | |
| Liver | 28.55 (4.86) | 31.03 (2.29) | | 20.17 (3.18) | | 22.16 (2.20) | |
| Kidney | 10.74 (2.03) | 9.48 (1.28) | | 6.18 (0.84) | | 9.66 (0.18) | |
| Intestine | 1.94 (0.25) | 4.27 (0.88) | | 7.06 (1.67) | | 14.34 (2.35) | |
| Spleen | 9.73 (3.34) | 9.70 (3.03) | | 7.86 (1.11) | | 5.44 (0.49) | |
| Pancreas | 4.12 (0.62) | 3.09 (0.38) | | 1.74 (0.33) | | 2.67 (0.07) | |
| Heart | 9.48 (2.01) | 6.51 (0.77) | | 2.98 (0.63) | | 4.30 (0.14) | |
| Lung | 15.47 (4.14) | 9.67 (0.88) | | 5.31 (0.67) | | 5.14 (0.29) | |
| Stomach^†^ | 1.64 (0.41) | 4.54 (0.97) | | 10.28 (2.03) | | 6.63 (1.12) | |
| Brain | 0.37 (0.05) | 0.19 (0.02) | | 0.16 (0.03) | | 0.08 (0.00) | |
|  | | | | | | | |
| [^99m^Tc]SB2A | | | | | | | |
| Blood^‡^ | 14.28 (1.90) | 7.50 (0.51) | | 5.13 (0.57) | | 4.17 (0.24) | |
| Liver | 25.52 (1.67) | 29.35 (2.85) | | 27.04 (3.90) | | 24.95 (3.38) | |
| Kidney | 5.03 (0.52) | 3.57 (0.41) | | 3.61 (0.29) | | 3.39 (0.20) | |
| Intestine | 1.52 (0.25) | 1.71 (0.11) | | 2.64 (0.36) | | 4.05 (0.55) | |
| Spleen | 10.76 (0.96) | 11.24 (2.26) | | 8.91 (3.26) | | 7.58 (2.60) | |
| Pancreas | 3.35 (0.60) | 2.12 (0.08) | | 2.06 (0.11) | | 1.83 (0.07) | |
| Heart | 12.64 (1.59) | 6.57 (0.92) | | 8.58 (1.32) | | 8.27 (1.19) | |
| Lung | 14.35 (1.86) | 7.79 (0.50) | | 7.26 (1.02) | | 6.48 (0.49) | |
| Stomach^†^ | 3.15 (0.56) | 8.78 (1.32) | | 16.13 (2.33) | | 14.72 (4.51) | |
| Brain^‡^ | 0.28 (0.03) | 0.17 (0.02) | | 0.12 (0.01) | | 0.11 (0.01) | |
|  | | | | | | | |
| [^99m^Tc]BT1B | | | | | | | |
| Blood | 18.61 (2.47) | 9.34 (2.62) | | 5.84 (0.65) | | 3.51 (0.34) | |
| Liver | 19.90 (4.06) | 25.64 (6.77) | | 27.06 (2.49) | | 19.04 (3.61) | |
| Kidney | 5.94 (0.73) | 4.29 (1.25) | | 5.24 (0.59) | | 3.20 (0.45) | |
| Intestine | 0.90 (0.25) | 2.00 (0.86) | | 6.81 (2.03) | | 9.95 (2.65) | |
| Spleen | 3.83 (0.84) | 3.00 (0.82) | | 3.11 (0.48) | | 1.36 (0.54) | |
| Pancreas | 1.80 (0.28) | 1.58 (0.55) | | 1.72 (0.11) | | 1.11 (0.18) | |
| Heart | 5.25 (0.22) | 3.30 (1.02) | | 2.72 (0.28) | | 1.64 (0.32) | |
| Lung | 10.87 (2.06) | 6.50 (1.41) | | 5.00 (0.34) | | 2.78 (0.54) | |
| Stomach^†^ | 1.39 (0.21) | 3.76 (0.82) | | 8.24 (1.41) | | 6.47 (0.85) | |
| Brain | 0.36 (0.03) | 0.17 (0.04) | | 0.11 (0.02) | | 0.08 (0.02) | |
|  | | | | | | | |
| [^99m^Tc]BT2B | | | | | | | |
| Blood | 23.15 (4.78) | 10.05 (1.78) | | 3.72 (0.49) | | 1.81 (0.44) | |
| Liver | 23.21 (5.72) | 33.78 (6.07) | | 31.45 (3.39) | | 35.76 (3.34) | |
| Kidney | 6.04 (0.96) | 5.02 (0.40) | | 3.47 (0.57) | | 3.13 (0.48) | |
| Intestine | 1.32 (0.21) | 1.97 (0.26) | | 6.44 (1.04) | | 13.90 (1.52) | |
| Spleen | 10.83 (1.58) | 14.49 (3.50) | | 10.91 (1.59) | | 4.80 (0.63) | |
| Pancreas | 2.81 (0.35) | 2.19 (0.35) | | 1.14 (0.25) | | 1.27 (0.28) | |
| Heart | 9.48 (4.83) | 8.62 (2.34) | | 6.67 (1.52) | | 7.08 (1.34) | |
| Lung | 22.06 (3.09) | 11.97 (1.01) | | 5.38 (1.16) | | 4.50 (0.60) | |
| Stomach^†^ | 0.69 (0.12) | 3.77 (0.56) | | 6.47 (1.37) | | 4.20 (1.21) | |
| Brain | 0.37 (0.11) | 0.20 (0.04) | | 0.10 (0.02) | | 0.05 (0.01) | |

*Expressed as % injected dose per gram. Each value is the mean (standard deviation) of 5 animals. ^†^Expressed as % injected dose per organ. ^‡^Data from our previous article (Ref. 35).

***In vitro* autoradiography of human CAA brain sections.** Postmortem brain tissues from autopsy-confirmed cases of CAA (female, 67 years old, and female, 85 years old) and a control (male, 73 years old) were obtained from the Graduate School of Medicine, Kyoto University, National Cerebral and Cardiovascular Center, and BioChain Institute Inc. (California, USA), respectively. Experiments were performed according to the regulations of the ethics committee of Kyoto University. Six micrometer thick serial sections of paraffin-embedded blocks were used for autoradiography. To completely deparaffinize the sections, they were incubated in xylene for 30 min two times and in 100% EtOH for 1 min two times. Subsequently, they were subjected to 1-min incubation in 90% EtOH and 1-min incubation in 70% EtOH, followed by a 5-min wash in water. Each slide was incubated with a 50% EtOH solution of [^99m^Tc]SB2A or [^99m^Tc]BT2B (370 kBq/mL) at room temperature for 1 h. For blocking experiments, the adjacent sections were incubated with a 50% EtOH solution of [^99m^Tc]BT2B (370 kBq/mL) in the presence of nonradioactive PIB (1.0 mM). The sections were washed in 50% EtOH for 3 min two times and exposed to a BAS imaging plate (Fuji Film, Tokyo, Japan) for 2 h. Autoradiographic images were obtained using a BAS5000 scanner system (Fuji Film). After autoradiographic examination, the same sections were immunostained by an antibody against Aβ(1−40) to confirm the presence of Aβ depositions. For immunohistochemical staining of Aβ(1−40), the sections were autoclaved for 15 min in 0.01 M citric acid buffer (pH 6.0) to activate the antigen. After three 5-min incubations in PBS-Tween 20 (PBST), they were incubated with anti-Aβ(1−40) primary antibody (BA27; Wako, Osaka, Japan) at room temperature overnight. Subsequently, they were incubated in PBST for 5 min three times, and incubated with biotinylated goat anti-mouse IgG (Wako) at room temperature for 3 h. After three 5-min incubations in PBST, the sections were incubated with Streptavidin-Peroxidase complex at room temperature for 30 min. After three 5-min incubations in PBST, they were incubated with diaminobenzidine (Merck, Hesse, Germany) as a chromogen for 5 min. After washing with water, the sections were observed under a microscope (BIOREVO BZ-9000; Keyence Corp., Osaka, Japan).

**Figure S1.** *In vitro* autoradiogram of a brain section from a patient with CAA (female, 67 years old) labeled with [^99m^Tc]BT2B (A). The same brain section was immunostained with an antibody against Aβ(1−40) (B). Blocking study with nonradioactive PIB was also performed using the adjacent brain section (C). *In vitro* autoradiogram of a brain section from a healthy control (male, 73 years old) labeled with [^99m^Tc]BT2B (D).

**Figure S2.** *In vitro* autoradiograms of brain sections from a patient with CAA (female, 85 years old) labeled with [^99m^Tc]SB2A (A) and [^99m^Tc]BT2B (B). Panel C and E represent magnified image details of panel A and B, respectively. The adjacent brain section was immunostained with an antibody against Aβ(1−40) (D). Red arrows indicate Aβ depositions labeled with both the bivalent ^99m^Tc-Ham complex and anti- Aβ(1−40) antibody.

***Ex vivo* autoradiography using Tg2576 and wild-type mice.** Tg2576 transgenic mice (female, 29 months old) and wild-type mice (female, 29 months old) were used as the Alzheimer’s model and age-matched control, respectively. A saline solution (150 μL) of [^99m^Tc]BT2B (18.5 MBq) containing ethanol (30 μL) was injected through the tail vein. The mice were sacrificed at 30 min postinjection. The brains were immediately removed, embedded in carboxymethylcellulose solution and then frozen in a dry ice/hexane bath. Sections of 30 μm were cut and exposed to a BAS imaging plate (Fuji Film) overnight. Autoradiographic images were obtained using a BAS5000 scanner system (Fuji Film). After autoradiographic examination, the same sections were stained by thioflavin-S to confirm the presence of Aβ depositions. For thioflavin-S fluorescent staining, the sections were immersed in a 100 μM thioflavin-S solution containing 50% EtOH for 3 min, washed in 50% EtOH for 1 min two times, and examined using a microscope (Keyence Corp.) equipped with a GFP-BP filter set. Additionally, the same sections were immunostained by anti-CD31 antibody to confirm the presence of endothelial cells. For immunohistochemical staining of CD31, the sections were incubated in PBST for 5 min three times, and incubated with anti-CD31 primary antibody (SZ31; Abcam, Cambridgeshire, U.K., dilution 1:50) at room temperature overnight. After three 5-min incubations in PBST, anti-rabbit secondary antibody (Dako, California, USA) incubation was carried out at room temperature for 3 h. Subsequently, the sections were incubated in PBST for 5 min three times, and incubated with diaminobenzidine (Merck) as a chromogen for 5 min. After washing with water, the sections were observed under a microscope (Keyence Corp.).

**Figure S3.** *Ex vivo* autoradiograms from Tg2576 (A) and wild-type (C) mice with [^99m^Tc]BT2B. The same sections were stained with thioflavin-S (D and F). The same sections were also immunostained with an antibody against CD31 (G and I). Panel B, E, and H represent magnified image details of panel A, D, and G, respectively. Red arrows indicate Aβ depositions labeled with both thioflavin-S and anti-CD31 antibody. White arrowheads indicate Aβ depositions labeled with thioflavin-S, not anti-CD31 antibody.

**SPECT/CT imaging.** Tg2576 transgenic mice (female, 27 or 30 months old) and wild-type mice (female, 27 or 30 months old) were used as the Alzheimer’s model and age-matched control, respectively. A saline solution (200 μL) of [^99m^Tc]SB2A (29.6−55.5 MBq) containing EtOH (40 μL) was injected directly into the tail vein. At 30 or 120 min postinjection, SPECT and CT images with the brain region as the ﬁeld of view were collected using the U-SPECT-II/CT system (MILabs, Utrecht, Netherlands) with 0.6-mm pinhole collimators (SPECT conditions, 30 min × 2 frames; CT conditions, accurate full angle mode in 65 kV/615 µA). The mouse was maintained under isoflurane anesthesia, while SPECT and then CT image data were collected. SPECT images were reconstructed by the OSEM method (16 subsets, 6 iterations) with a 0.4-mm Gaussian ﬁlter.


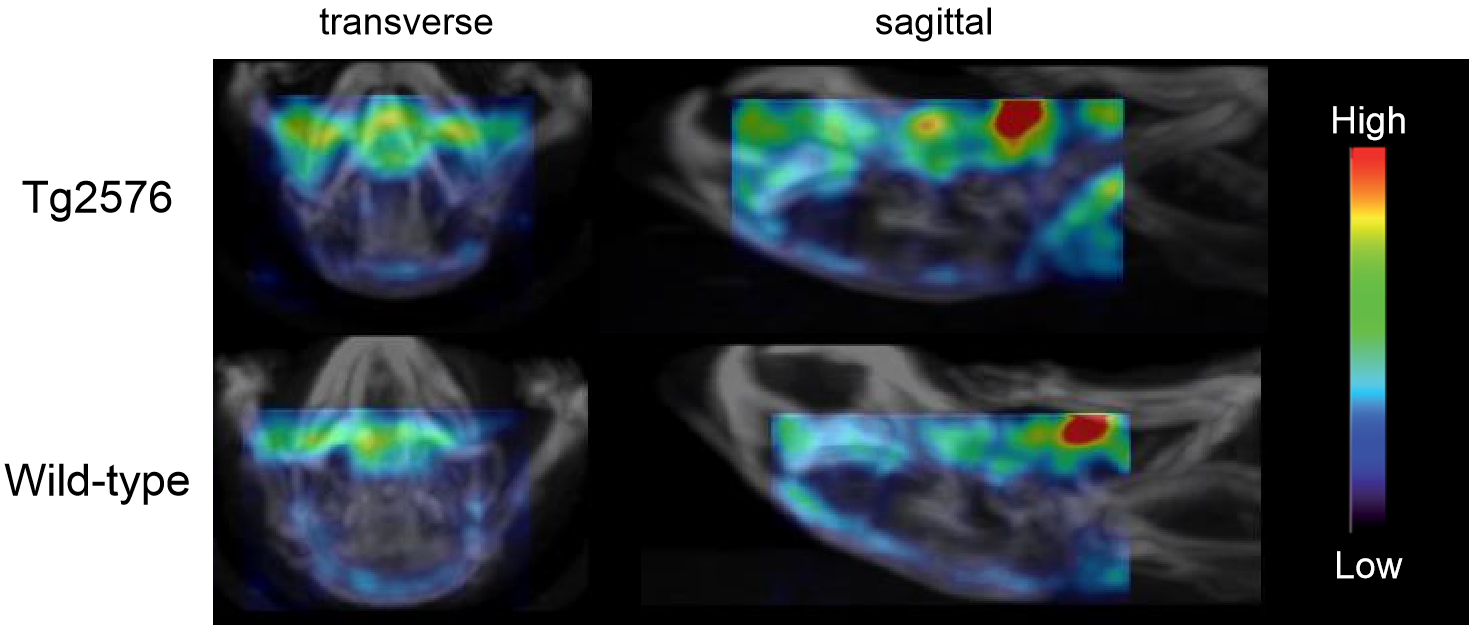


**Figure S4.** SPECT/CT imaging of [^99m^Tc]SB2A in Tg2576 and wild-type mice at 30 min postinjection.


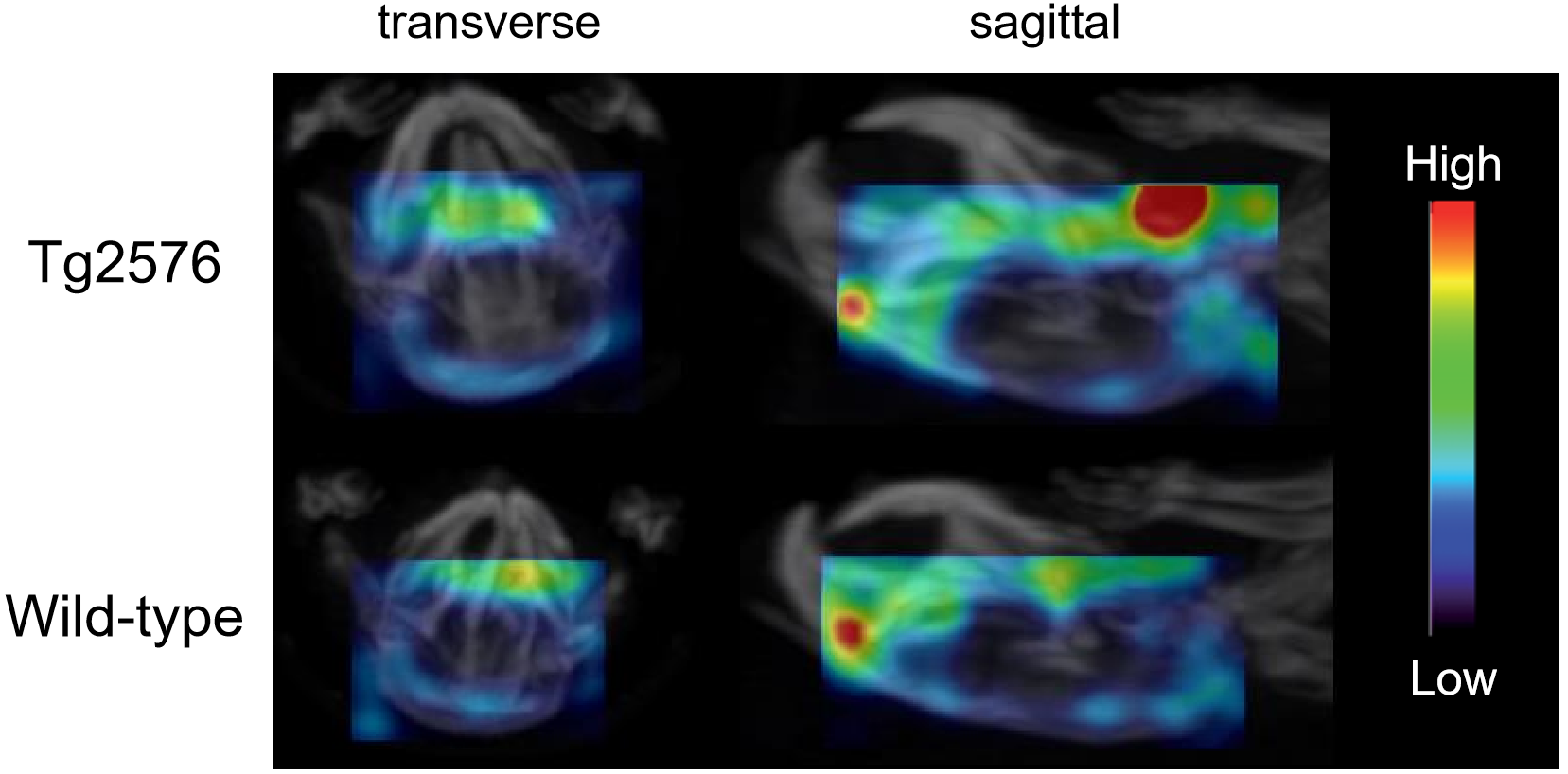


**Figure S5.** SPECT/CT imaging of [^99m^Tc]SB2A in Tg2576 and wild-type mice at 120 min postinjection.
